# Supplementary material for: Predicting the Printability in Selective Laser Melting with a Supervised Machine Learning Method
Source: Materials (Basel). 2020 Nov 10;13(22):5063. doi: 10.3390/ma13225063 (PMC7698234; doi:10.3390/ma13225063)
Supplement: Supplementary file 1 [file materials-13-05063-s001.pdf]

materials

MDPI

Supplementary material

Predicting the Printability in Selective Laser Melting with a Supervised Machine Learning Method

Yingyan Chen [1,2], Hongze Wang [1,2*], Yi Wu [1,2*] and Haowei Wang [1,2]

[1] State Key Laboratory of Metal Matrix Composites, Shanghai Jiao Tong University, No. 800 Dongchuan Road, Shanghai 200240, China; cyy1996@sjtu.edu.cn (Y.C.); hwwang@sjtu.edu.cn (H.W.)

[2] School of Materials Science and Engineering, Shanghai Jiao Tong University, No. 800 Dongchuan Road, Shanghai 200240, China

* Correspondence: hz.wang@sjtu.edu.cn (H.W.); eagle51@sjtu.edu.cn (Y.W.)

Table S1. The values of four evaluation indicators for all the parameter combinations.

Laser Power W	Scan Speed mm/s	Input Variables			
		$\overline{w}$ μm	$\overline{h}$ μm	R_w %	R_h %
300	2200	84.72	23.82	28.13	52.44
300	2000	101.54	25.48	20.40	46.92
300	1800	101.37	32.28	15.56	30.52
300	1600	121.44	35.46	11.40	26.74
300	1400	135.40	37.59	6.43	16.70
300	1200	138.70	42.23	5.81	18.40
300	1000	157.60	47.09	5.44	16.97
300	800	176.54	41.77	4.42	16.82
300	600	190.58	39.01	3.20	15.43
300	400	233.42	26.76	1.83	15.29
300	200	251.70	20.16	1.63	15.91
195	2200	73.43	13.49	34.91	57.18
195	2000	81.92	15.38	24.64	43.85
195	1800	92.32	19.97	11.69	26.80
195	1600	99.27	22.89	8.88	21.70
195	1400	106.43	26.05	8.68	19.35
195	1200	122.63	29.41	8.04	17.75
195	1000	127.82	32.31	7.41	17.47
195	800	142.33	33.95	6.56	18.59
195	600	160.40	35.46	5.18	19.69
195	400	172.15	32.98	4.10	19.33
195	200	188.75	27.95	3.65	19.10
90	2200	23.36	10.75	69.32	78.17
90	2000	24.45	11.89	69.65	77.79
90	1800	34.21	15.81	67.12	73.62
90	1600	34.86	18.87	65.25	74.30
90	1400	36.39	24.09	66.15	73.42
90	1200	44.17	25.65	62.22	72.63
90	1000	50.11	26.77	60.24	70.36
90	800	78.20	28.41	45.60	65.13
90	600	101.80	30.56	25.13	55.88
90	400	106.06	35.30	17.31	48.31
90	200	130.88	30.39	14.37	40.91

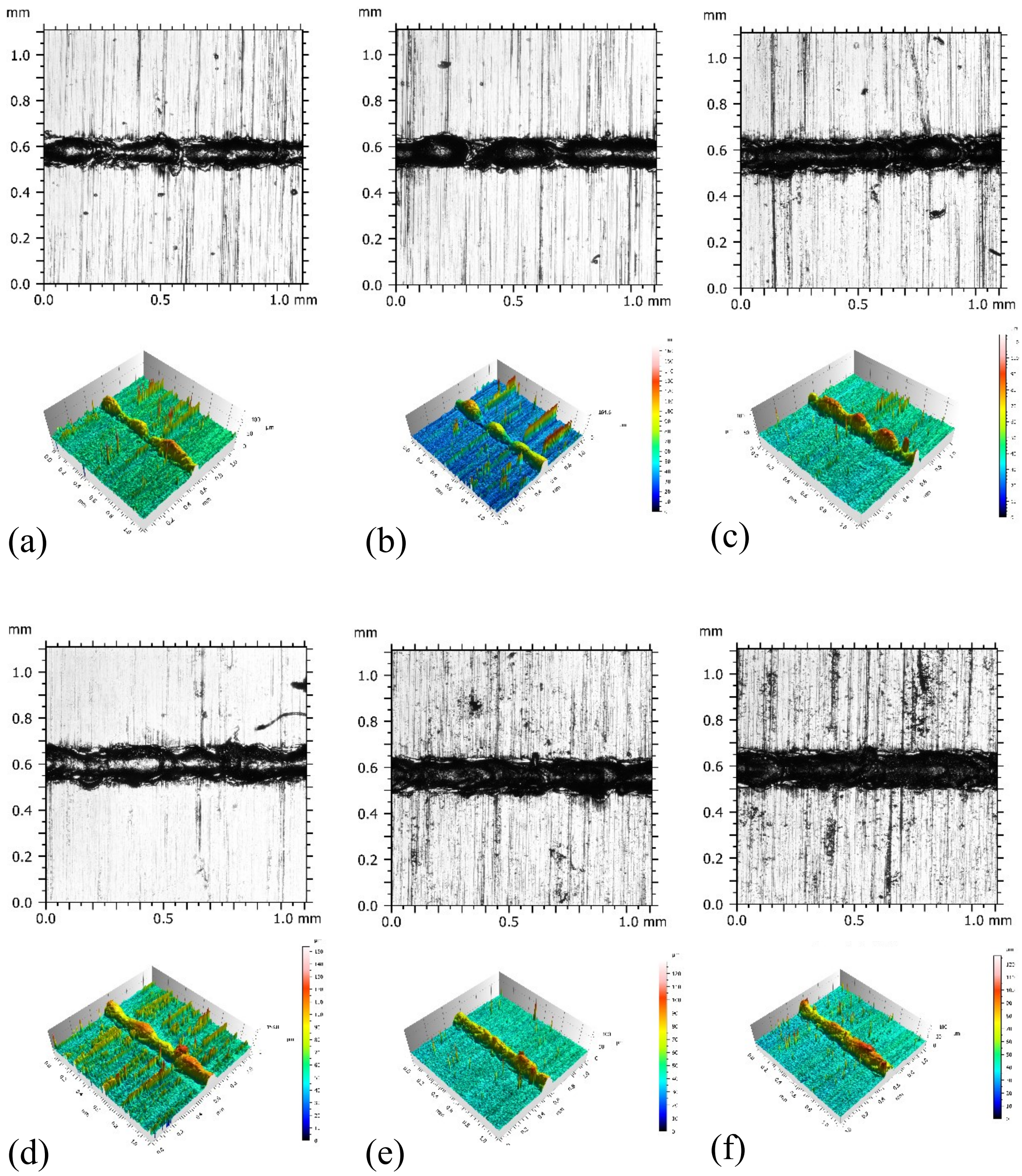

Figure S1. The surface morphology of SLM-fabricated single tracks from TiB_2/AlSi10Mg powder on Al alloy substrate (all at the same magnification). (**a**) P = 300 W and V = 2200 mm/s; (**b**) P = 300 W and V = 2000 mm/s; (**c**) P = 300 W and V = 1800 mm/s; (**d**) P = 300 W and V = 1600 mm/s; (**e**) P = 300 W and V = 1400 mm/s; (**f**) P = 300 W and V = 1200 mm/s. .

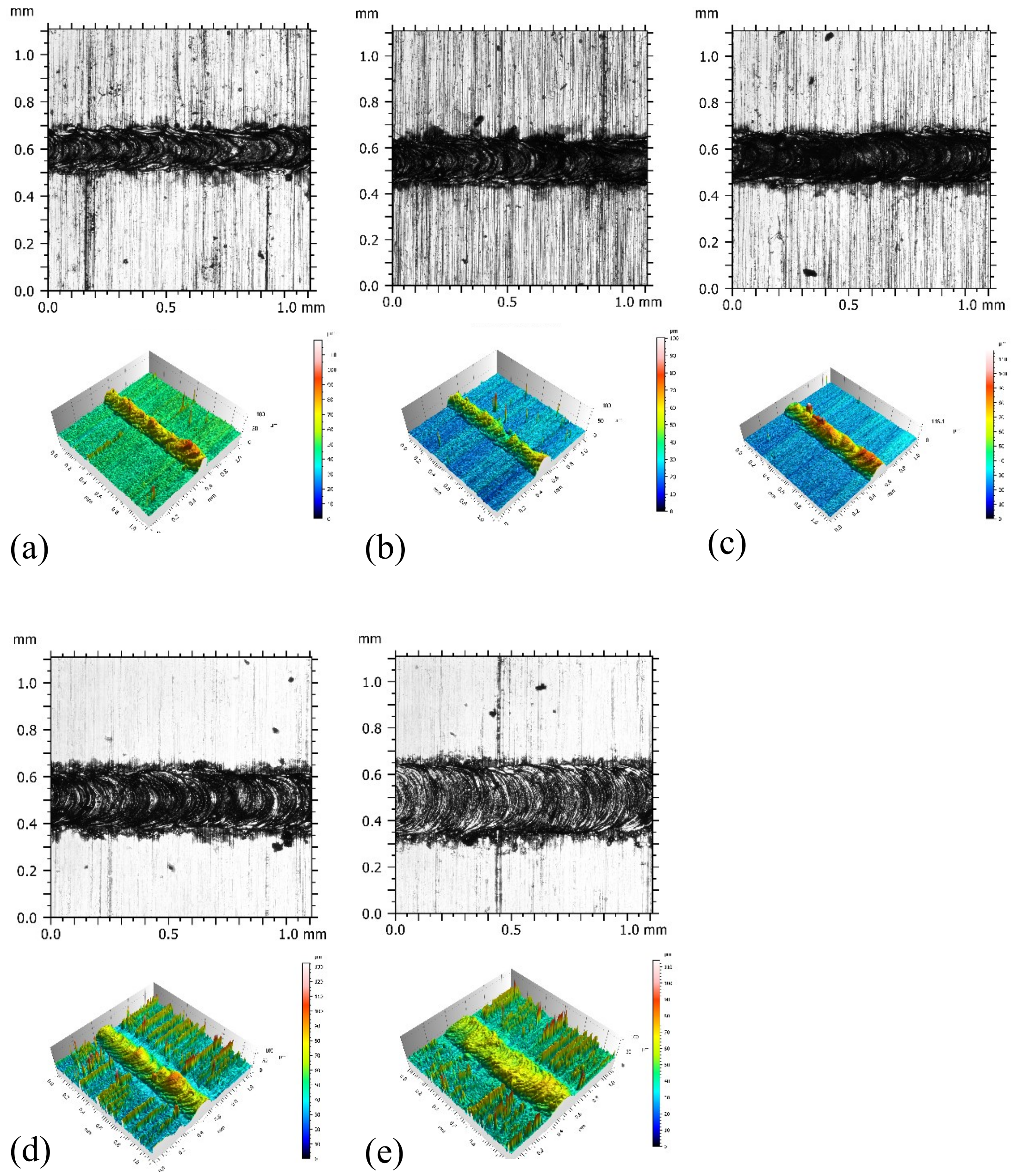

Figure S2. The surface morphology of SLM-fabricated single tracks from TiB_2/AlSi10Mg powder on Al alloy substrate(all at the same magnification). (**a**) P = 300 W and V = 1000 mm/s; (**b**) P = 300 W and V = 800 mm/s; (**c**) P = 300 W and V = 600 mm/s; (**d**) P = 300 W and V = 400 mm/s; (**e**) P = 300 W and V = 200 mm/s.

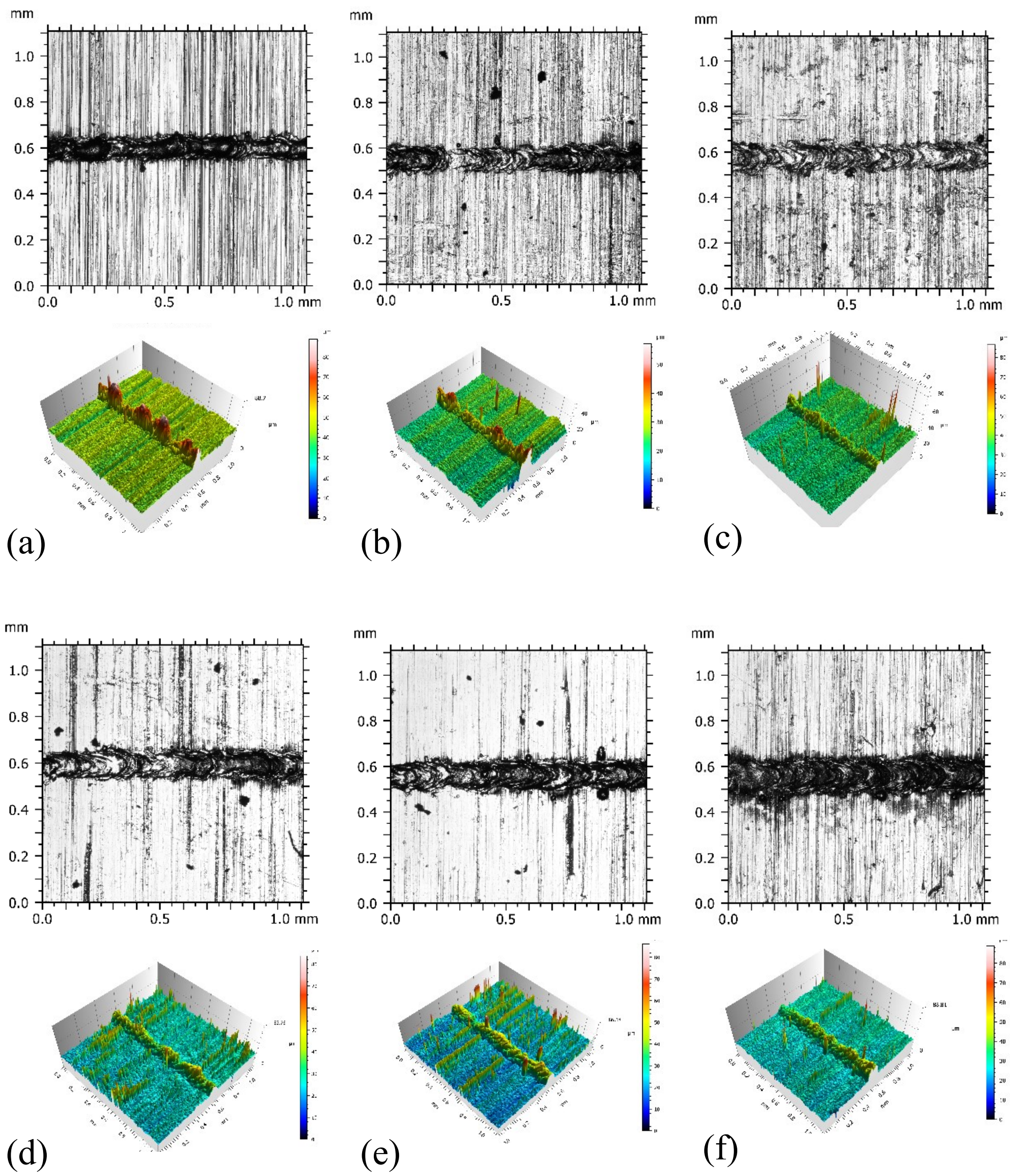

Figure S3. The surface morphology of SLM-fabricated single tracks from TiB_2/AlSi10Mg powder on Al alloy substrate (all at the same magnification). (**a**) P = 195 W and V = 2200 mm/s; (**b**) P = 195 W and V = 2000 mm/s; (**c**) P = 195 W and V = 1800 mm/s; (**d**) P = 195 W and V = 1600 mm/s; (**e**) P = 195 W and V = 1400 mm/s; (**f**) P = 195 W and V = 1200 mm/s.

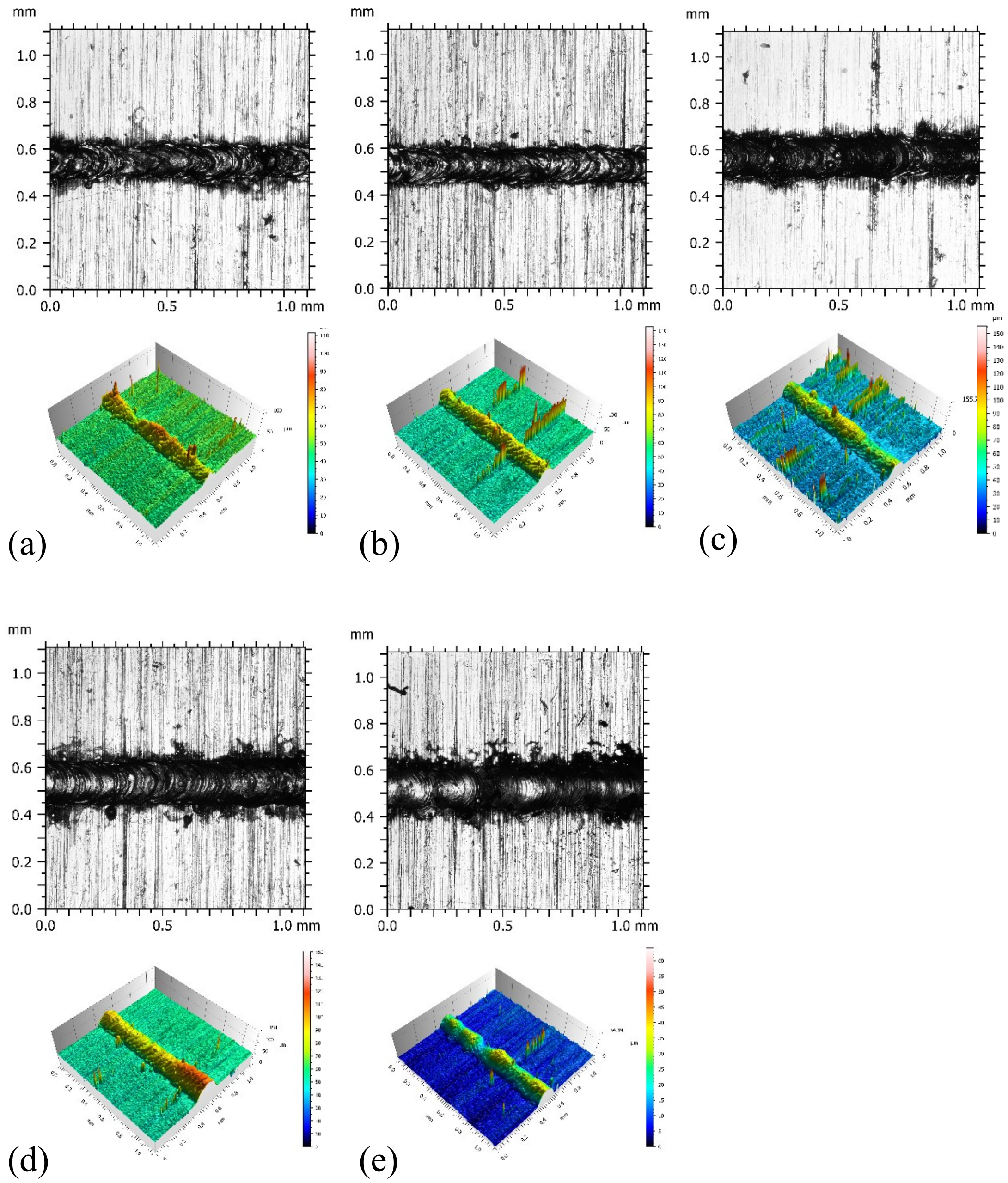

Figure S4. The surface morphology of SLM-fabricated single tracks from TiB_2/AlSi10Mg powder on Al alloy substrate(all at the same magnification). (**a**) P = 195 W and V = 1000 mm/s; (**b**) P = 195 W and V = 800 mm/s; (**c**) P = 195 W and V = 600 mm/s; (**d**) P = 195 W and V = 400 mm/s; (**e**) P = 195 W and V = 200 mm/s.

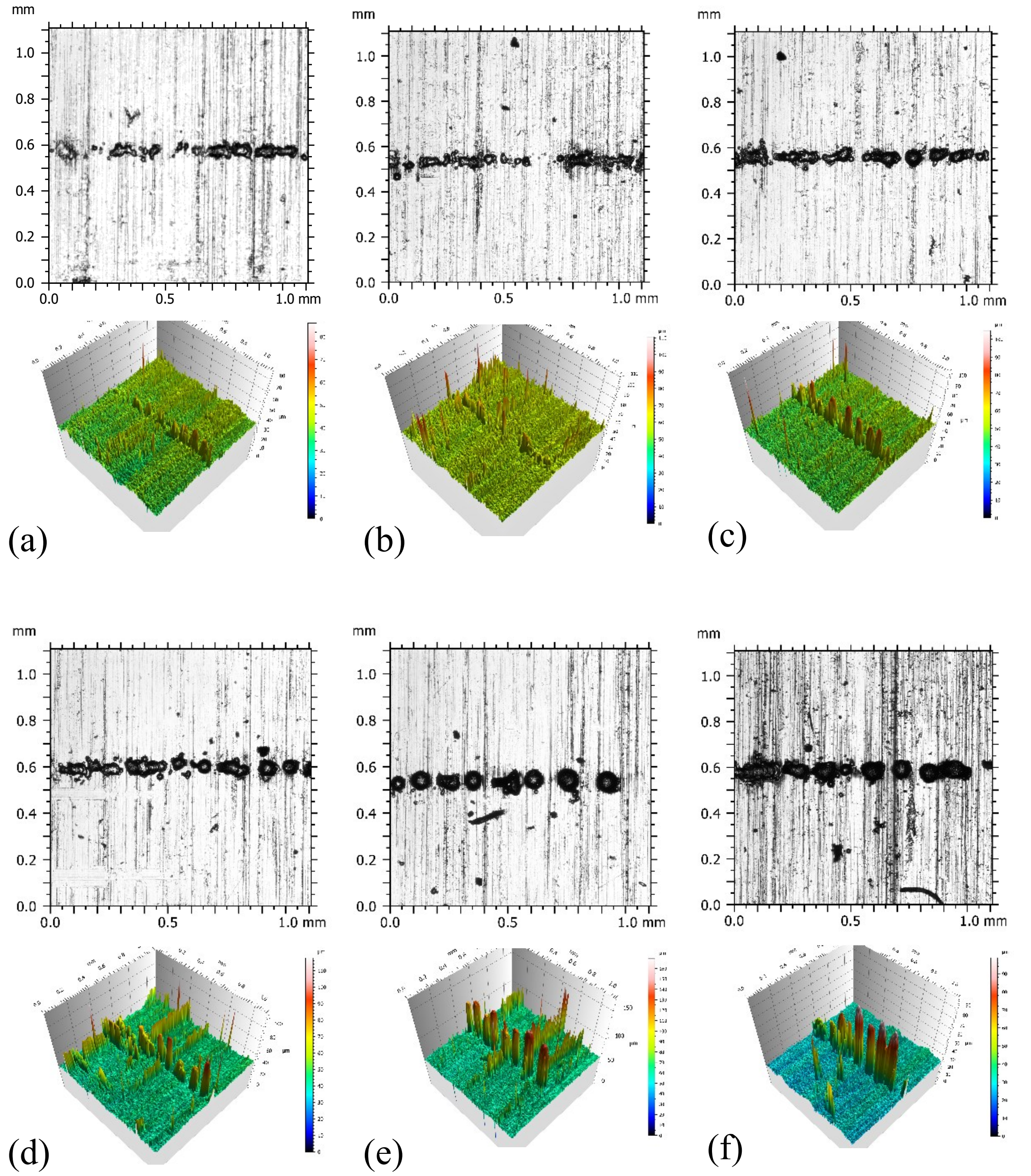

Figure S5. The surface morphology of SLM-fabricated single tracks from TiB_2/AlSi10Mg powder on Al alloy substrate(all at the same magnification). (a) P = 90 W and V = 2200 mm/s; (b) P = 90 W and V = 2000 mm/s; (c) P = 90 W and V = 1800 mm/s; (d) P = 90 W and V = 1600 mm/s; (e) P = 90 W and V = 1400 mm/s; (f) P = 90 W and V = 1200 mm/s.

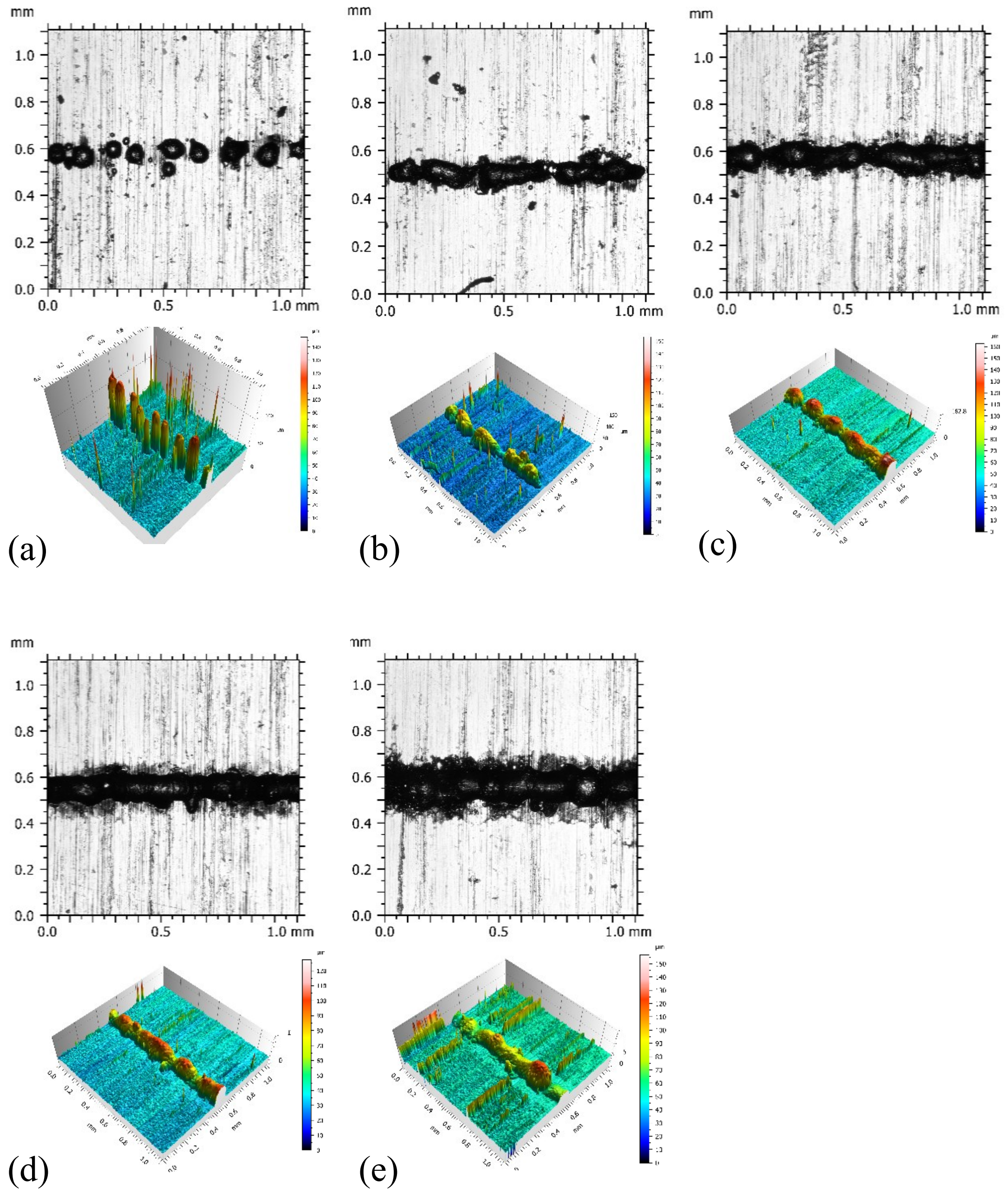

Figure S6. The surface morphology of SLM-fabricated single tracks from TiB_2/AlSi10Mg powder on Al alloy substrate(all at the same magnification). (**a**) P = 90 W and V = 1000 mm/s; (**b**) P = 90 W and V = 800 mm/s; (**c**) P = 90 W and V = 600 mm/s; (**d**) P = 90 W and V = 400 mm/s; (**e**) P = 90 W and V = 200 mm/s.
